# Neuroendocrine Alterations in Obese Patients with Sleep Apnea Syndrome

**DOI:** 10.1155/2010/474518

**Published:** 2010-02-23

**Authors:** Fabio Lanfranco, Giovanna Motta, Marco Alessandro Minetto, Matteo Baldi, Marcella Balbo, Ezio Ghigo, Emanuela Arvat, Mauro Maccario

**Affiliations:** Division of Endocrinology, Diabetology and Metabolism, Department of Internal Medicine, University of Turin, Corso Dogliotti 14, 10126 Torino, Italy

## Abstract

Obstructive sleep apnea syndrome (OSAS) is a serious, prevalent condition that has significant morbidity and mortality when untreated. It is strongly associated with obesity and is characterized by changes in the serum levels or secretory patterns of several hormones. Obese patients with OSAS show a reduction of both spontaneous and stimulated growth hormone (GH) secretion coupled to reduced insulin-like growth factor-I (IGF-I) concentrations and impaired peripheral sensitivity to GH. Hypoxemia and chronic sleep fragmentation could affect the sleep-entrained prolactin (PRL) rhythm. A disrupted Hypothalamus-Pituitary-Adrenal (HPA) axis activity has been described in OSAS. Some derangement in Thyroid-Stimulating Hormone (TSH) secretion has been demonstrated by some authors, whereas a normal thyroid activity has been described by others. Changes of gonadal axis are common in patients with OSAS, who frequently show a hypogonadotropic hypogonadism. Altogether, hormonal abnormalities may be considered as adaptive changes which indicate how a local upper airway dysfunction induces systemic consequences. The understanding of the complex interactions between hormones and OSAS may allow a multi-disciplinary approach to obese patients with this disturbance and lead to an effective management that improves quality of life and prevents associated morbidity or death.

## 1. Introduction

Obstructive sleep apnea syndrome (OSAS) is an emerging public health problem and is characterized by repetitive upper airway occlusion episodes leading to apnea and asphyxia, typically occurring 100–600 times per night, with arousal being required to reestablish airway patency [1–3].

The most important epidemiological risk factors for sleep apnea are obesity and male gender [2, 4, 5]. OSAS is estimated to affect up to 7% of the adult male population, and its prevalence increases with advancing age, although the clinical severity of apnea decreases [4–6]. The increased risk of sleep apnea in male subjects may be due to differences in airway anatomy, pharyngeal dilator muscle function, and ventilatory control mechanisms [4, 5].

The pathophysiology of OSAS is complex and incompletely understood but is mainly based on an imbalance between the collapsing forces of the upper airway during inspiration and the counteracting forces of the upper airway dilating muscles [7, 8]. 

The sequence of events in an episode of apnea consists of upper airway constriction, progressive hypoxemia secondary to asphyxia, autonomic and EEG arousal sufficient to prompt one to open and clear the airway to reverse the asphyxia, followed by successive relaxation of the airway, upper airway constriction, and so forth. As the cycle repeats itself throughout the night, the patient's sleep is fragmented [8, 9]. Daytime sleepiness results, along with decreased cognitive performance, decreased quality of life and an increased risk of industrial and motor vehicle accidents [2, 4, 5]. Moreover, OSAS may increase the risk of developing some of the features of the metabolic syndrome, including hypertension, insulin resistance, and type 2 diabetes, leading to adverse cardiovascular consequences such as myocardial infarction and stroke [8, 10–13]. Thus, more than a local abnormality, OSAS should be considered a systemic disease [13].

Because the treatment of OSAS provides many benefits to patients and society, it is very important to obtain an early diagnosis. The diagnosis of OSAS is based on the combination of clinical features and compatible findings on instrumental tests in which multiple physiologic signals are monitored simultaneously during a night of sleep. Polysomnography is routinely indicated for measuring sleep stages and sleep-disordered breathing, for detection of OSAS [14–16]. The severity of OSAS is commonly expressed as the apnea/hypopnea index (AHI), which indicates the frequency of the apnea/hypopnea episodes per hour of sleep [2, 3, 17]. 

Nasal continuous positive airway pressure (nCPAP) is the initial treatment of choice for most patients because of its noninvasive approach and technical efficacy. It is worn on a nightly basis and acts like a constant pressure air splint to prevent collapse of the upper airway during sleep. nCPAP provides immediate relief of symptoms and has only minor side effects [18]. Nevertheless, an alternative treatment is needed if nCPAP is not feasible for medical or psychological reasons. Removable oral appliances that advance the mandible when fitted to the teeth during sleep also improve nocturnal breathing disturbances, symptoms, quality of life, and vigilance in OSAS patients. However, their long-term effectiveness and side effects require further study [19]. In morbidly obese patients suffering from OSAS bariatric surgery should be considered as a treatment that reduces obesity and at the same time improves OSAS [20]. In selected patients including those with adeno-tonsillar hypertrophy, and craniofacial malformations various surgical techniques that enlarge the upper airway may be a treatment option for OSAS, although the effectiveness in improving OSAS and snoring has been inconsistent and unpredictable [19, 21, 22].

An accumulating body of evidence shows that OSAS induces changes in the serum levels or secretory patterns of several hormones. These changes are not only related to obesity but may be due also to sleep fragmentation induced by apnea and hypopnea, to frequent arousals leading to an increase in stress hormone levels [23], to direct effects of hypoxia on central neurotransmitters with alterations in the hypothalamus-pituitary axes and in the secretion of the peripheral endocrine glands [24], and to the effects of hypercapnia which may increase levels of adrenocorticotropic hormone (ACTH) and adrenal hormones [9, 25]. Finally, the sleep pattern disruption, sleep loss, and naps cause an alteration of hormonal circadian rhythms. 

In this review the neuroendocrine changes associated to OSAS in obesity will be revised. Namely, alterations of the growth hormone (GH)/insulin-like growth factor-I (IGF-I), adrenal, thyroid, and gonadal axes will be discussed.

## 2. Obstructive Sleep Apnea Syndromeand Obesity

Several clinical and epidemiological studies have demonstrated the strict association existing between OSAS and obesity. The incidence of OSAS among morbidly obese patients is 12- to 30-fold higher than in the general population [26]. Body mass index (BMI) has been shown to be the most important risk factor for OSAS, preceding age and male gender. In particular, the android-central type of obesity is strictly associated to OSAS. Neck circumference is the most important predictor of OSAS among obese subjects [4].

Several mechanisms are involved in the association of obesity and OSAS. These include upper airway alterations such as an increased collapsibility of the peripharyngeal tissues. The accumulation of adipose tissue in the neck and also in the pharyngeal structures induces an airway restriction and collapse during inspiration [19, 27]. Abnormalities in the chest wall dynamics, a reduced respiratory compliance, and an impaired respiratory muscle function contribute to the pulmonary dysfunction of severely obese patients [28]. 

Noteworthy, OSAS itself may predispose individuals to worsening obesity because of sleep deprivation, daytime somnolence, and disrupted metabolism [12, 18]. 

Weight reduction has been proven effective in reducing the severity of the sleep disturbance, probably through an influence on the upper airways structure and function [20, 29].

It is well known that simple obesity is associated with coronary heart disease (CHD) and is an established marker of cardiovascular risk. Obstructive sleep apnea is an independent risk factor for hypertension [10, 30] and is thought to be a cause of premature death, ischaemic heart disease, and stroke [13]. Moreover, OSAS is connoted by a specific worsening in endocrine and metabolic abnormalities which can account for a further increase in cerebro- and cardiovascular risk [13]. 

It is also well established that sleep deprivation has an impact on glucose metabolism [8, 11–13, 31]. A growing body of epidemiological evidence supports an association between chronic partial sleep deprivation and the risk for obesity, insulin resistance, and diabetes [31]. Because OSAS is associated with sleep fragmentation, effectively sleep loss, and daytime sleepiness, the insulin sensitivity in patients has been assessed and indeed insulin resistance has been reported [11, 32, 33] (Table 1). That OSAS is an independent risk factor for increased insulin resistance can be learned from improvement in insulin sensitivity after 3 months of treatment with nCPAP [34].

Recently, adipocytokines, adipocyte-specific secreted proteins, have been considered to play an important role in the progression of atherosclerotic disease in obesity and in OSAS. Of all adipocytokines, leptin and adiponectin have received the most attention. Despite the antiobesity effect of leptin, obese subjects often have increased serum leptin levels [35]. In contrast, adiponectin levels are decreased in obese individuals and are associated with insulin resistance and hyperinsulinemia [36, 37] (Table 1). 

Serum leptin levels have been shown to be positively correlated with the severity of OSAS in obese subjects [37–39], suggesting that leptin is a hormonal factor affected by OSAS. However, Barceló et al. [40] did not observe a leptin decrease in obese OSAS during prolonged nCPAP treatment and recorded only a slight reduction in nonobese OSAS, speculating that the increased leptin levels described so far in patients with OSAS are mostly associated with obesity and not with the disease itself. Similarly, a correlation between adiponectin concentrations and the severity of OSAS have been described by some [41, 42] but not by other authors [37] and nCPAP failed to increase adiponectin concentrations in obese patients with OSAS [43] (Table 1).

Interestingly, apart its antiobesity effects, leptin exerts important physiological effects on the control of respiration and has been suggested to be a better predictor than percent body fat for the presence of hypercapnia in patients with obesity-hypoventilation syndrome [44].

Ghrelin is a hormone that influences appetite and fat accumulation and its physiological effects are opposite to those of leptin. No clear relation has been found between ghrelin and OSAS (Table 1). In a study of 30 obese OSAS patients, plasma ghrelin levels were significantly higher in OSAS patients than in controls and rapidly decreased with nCPAP therapy, suggesting that the elevated ghrelin levels could not have been determined by obesity alone [45]. The appetite stimulating effects of ghrelin may contribute to increased caloric intake and weight gain in patients with OSAS [8]. In a study of 30 untreated obese patients with moderate-severe OSAS, significantly higher levels of serum leptin were found in OSAS patients than in controls, but ghrelin levels presented no such difference [39]. Thus, the relation between OSAS and ghrelin is still an unresolved issue.

## 3. Neuroendocrine Disturbances in Obstructive Sleep Apnea Syndrome

### 3.1. GH/IGF-I Axis

OSAS is connoted by a reduced spontaneous GH secretion, which seems to be due to the sleep respiratory disturbance and is restored by nCPAP treatment. Saini et al. [46] have shown that a single night nCPAP treatment is able to increase the duration of slow wave sleep and to normalize GH levels in obese subjects with OSAS. The treatment increases the amplitude but not the frequency of GH secretory bursts. These authors underlined the tight connection between sleep fragmentation and low circulating GH levels in untreated OSAS patients. Since no modification of the concomitant hyperinsulinemia was recorded after nCPAP treatment, they concluded that insulin did not play a role in the pathogenesis of GH reduction in these patients. 

Cooper et al. [47] confirmed the effects of nCPAP treatment on GH secretion in obese patients with OSAS and demonstrated an increase of FFA concentrations indicating an increase of GH peripheral lipolytic action. 

Reduced IGF-I levels have also been described in obese patients with OSAS [49]. This reduction is greater than in obese subjects without OSAS and is independent of BMI and age. These authors outlined the role of hypoxemia rather than sleep fragmentation in the pathogenesis of hormonal alterations and documented a significant increase in IGF-I levels three months after the initiation of nCPAP treatment. 

Ursavas et al. [50] recently demonstrated that the low circulating IGF-I levels in patients with OSAS were negatively correlated with AHI, duration of apnea-hypopnea, arousal index, average desaturation, and oxygen saturation index. These authors have also hypothesized that the negative correlation between obesity and IGF-I levels that was found in previous studies can be related to the presence of OSAS in the majority of obese patients.

We have demonstrated that obese patients with OSAS show an impairment of both basal and stimulated GH secretion [48]. In fact, in obese patients with OSAS we have found basal GH levels similar to those recorded in patients with simple obesity and lower than in normal subjects, with a deeper reduction of the GH response to a provocative test as potent and reproducible as GHRH plus arginine [72]. Interestingly, this impairment occurred in the presence of basal IGF-I levels significantly lower than in simple obesity and not responsive to the short-term administration of a very low recombinant human GH dose. These data support the hypothesis that OSAS per se may impair GH/IGF-I axis activity, independently of adiposity. Accordingly, the reduction of nocturnal GH secretion of overweight patients with OSAS is reversed by nCPAP, before any significant variation in body weight occurs [46, 47]. Moreover, nCPAP has been found able to restore IGF-I levels in OSAS patients independently of body weight variations [49].

The mechanisms controlling sleep and GH secretion are tightly associated [73], and the amplification of somatotroph secretion during sleep, for example, III-IV stages, is well known [74]. Qualitative and quantitative sleep alterations in OSAS have been clearly demonstrated [75] and are improved by nCPAP treatment, together with GH and IGF-I secretion [46, 49]. In particular, slow-wave sleep is specifically and markedly reduced or absent in OSAS [76]. Thus, sleep-related alterations of the neuroendocrine control of GH secretion could also contribute to the peculiar impairment of GH release in obese patients with OSAS.

**Figure 1:**
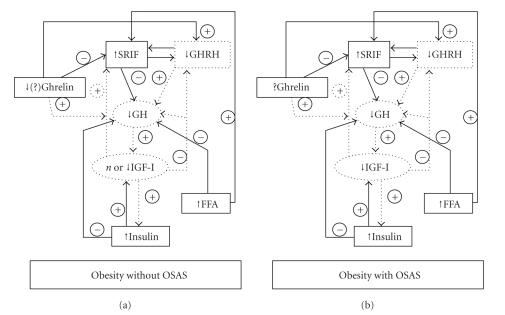
Regulation of GH secretion in obese patients without OSAS and obese patients with OSAS.

Insulin resistance can be also proposed to explain the impairment of GH/IGF-I axis activity in OSAS. In fact, insulin is able to inhibit GH synthesis and secretion [77] and to enhance GH-induced hepatic IGF-I production [78] (Figure 1). 

Finally, hypoxia itself might impair both somatotroph function and the peripheral sensitivity to GH. In fact, animal studies have shown that acute as well as prolonged hypoxia reduces GH synthesis and release [71]. Moreover, hypoxia reduces IGF-I mRNA expression in endothelial cells in vitro [79], and low IGF-I levels have been shown in patients with ischemic dilated cardiomyopathy [80]. 

In conclusion, obese patients with OSAS show a peculiar reduction of both spontaneous and stimulated GH secretion coupled to reduced IGF-I concentrations and impaired peripheral sensitivity to GH. These endocrine abnormalities can be responsible for metabolic alterations, which are common in OSAS and increase the risk of cardiovascular events as well as mortality.

### 3.2. Prolactin

Plasma prolactin (PRL) concentrations exhibit a sleep-dependent pattern, with highest levels during sleep and lowest levels during the waking period. In human physiology, outside pregnancy, PRL secretion is altered by increasing body weight in both children and adults [81]. PRL in this circumstance appears to be a marker of hypothalamus-pituitary function: the PRL response to insulin-hypoglycaemia, Thyrotropin-Releasing Hormone (TRH) stimulation, and other stimulatory factors may be diminished [81–83]. In addition, obesity alters the 24 hour spontaneous release of PRL with a generalised dampening of release. These alterations seem to be due to obesity per se with its hyperinsulinaemic state as well as an interplay between PRL and leptin concentrations. Weight reduction, with accompanying decrease in plasma insulin levels, leads to a normalization of PRL responses in most, but not all, circumstances [81].

Studies on PRL secretion axis in OSAS provided conflicting results [49, 51, 84]. Hypoxemia and chronic sleep fragmentation in OSAS could affect the sleep-entrained PRL rhythm [52]. PRL secretion is reversibly elevated in an hypoxic stress response and can be a useful marker of acute, severe, illness-induced stress [53]. No correlation of PRL levels with OSAS severity and no changes induced by nCPAP treatment have been described by Grunstein et al. [49] and by Meston et al. [53]. We have found similar basal PRL concentrations as well as PRL response to TRH in obese subjects with OSAS, in obese subjects without OSAS, and in normal lean controls [51]. On the other hand, Spiegel et al. [52] have described a lower PRL pulse frequency in untreated OSAS patients as compared to treated patients and have indicated that nCPAP treatment immediately restores a normal sleep structure and normalizes PRL release by restoring pulse frequency to values similar to those observed in normal subjects.

### 3.3. Adrenal Axis

A disrupted Hypothalamus-Pituitary-Adrenal (HPA) axis activity has been described in OSAS. Nocturnal awakenings are associated with pulsatile cortisol release [23] and autonomic activation. The latter leads to increased catecholamine release as well as Corticotropin-Releasing Hormone (CRH) and cortisol release. Sleep deprivation itself is associated with HPA axis hyperactivity [85]. 

The literature varies regarding the effects of OSAS on the HPA system. An enhanced cortisol secretion in patients with OSAS has been reported by some [54, 56, 57] but not by other authors [49, 53, 58, 59]. In a group of obese male patients with OSAS we have found normal adrenocorticotropic hormone (ACTH) and cortisol levels but an ACTH hyper-responsiveness to CRH that, in fact, was even more remarkable than that occurring in nonapneic obese patients [51]. This peculiar ACTH response pattern in obese OSAS patients indicates that factors other than obesity per se have a role in this clinical condition. In fact, a disrupted HPA axis function has been described in patients with abdominal obesity, including both neuroendocrine and peripheral alterations leading to inappropriately higher than normal exposure to cortisol of peripheral tissues, particularly the visceral adipose tissue. The disregulation of the HPA axis in abdominal obesity has been demonstrated also by many dynamic studies showing both a high cortisol secretion after laboratory stress test and a hyper-responsiveness after various provocative stimuli [86]. Altered HPA axis activity in obesity may also derive from a dysruption of the catecholaminergic regulation in the central nervous system, particularly during acute and chronic stress challenges [87].

Hypoxia is likely to directly or indirectly play a critical role in the alteration of the HPA axis activity in OSAS, since it has been shown able to induce HPA axis activation both in animals and in humans [88, 89]. In fact, an hypoxic state is likely to represent a stressful condition that, in turn, could well trigger the HPA axis. Moreover, sleep-related alterations of the neuroendocrine control of anterior pituitary function could contribute to the peculiar alteration of ACTH secretion in obese patients with OSAS.

Several studies have primarily focused on the effects of nCPAP on cortisol. Some authors have reported that nCPAP does not reduce cortisol levels [49] or that acute withdrawal of nCPAP therapy does not result in an increase in cortisol levels [90]. In contrast, other studies have reported that nCPAP does reverse hypercortisolemia [91], particularly with prolonged use [56]. Noteworthy, several of these studies were limited in that cortisol was measured at a single time point, and consequently they do not measure potential clinically important HPA axis and rhythm changes. Vgontzas et al. [55] have demonstrated that sleep apnea in obese men is associated with increased cortisol levels during the night, compared with nonapneic obese controls, which is corrected after the use of nCPAP for 3 months. The correction of the increased cortisol appears to be related to the elimination, through nCPAP, of the stress of repetitive respiratory pausing and sleep fragmentation and/or better oxygen saturation [55]. Finally, Carneiro et al. [60] have recently indicated that men with OSAS present a blunted response of cortisol suppression after dexamethasone, which is recovered after 3 months of nCPAP therapy. 

When untreated, this HPA axis hyperactivity in OSAS may be a risk factor for the metabolic syndrome, and also for insomnia and depression, which are both associated with hypercortisolemic states. Similar sleep EEG changes were found in depression and Cushing's disease, and a role for obstructive sleep apnea has been suggested [92].

Several components of the renin-angiotensin-aldosterone system (RAAS) are elevated in obese humans [61] and play an important role in the etiology of obesity hypertension [93]. In addition, plasma renin activity declines with weight loss and is correlated with the reduction in blood pressure [94, 95]. 

To our knowledge, there is a lack of data on the effects of OSAS on the mineralocorticoid axis, although hypertension is common in OSAS patients and may result from prolonged repetitive stimulation of the renin-aldosterone axis [96]. Moreover, it is well recognized that renin is elevated in response to systemic illness [97]. However, Meston et al. [53] did not find a relationship between OSAS severity and aldosterone or renin levels in overweight and obese OSAS patients. A recent report demonstrated that elevated aldosterone is a cause of hypertension in OSAS, but the cause of hyperaldosteronism was unknown [62]. Since ACTH stimulates both aldosterone and cortisol synthesis and secretion, it has been hypothesized that the HPA axis hyperactivity from OSAS may increase aldosterone and thereby contribute to hypertension [9]. This same mechanism has been proposed for explaining hypertension in depression [98].

### 3.4. Thyroid Axis

A link between OSAS and hypothyroidism is suggested by the high prevalence of sleep apnea among hypothyroid patients, particularly in rare myxoedematous patients [99, 100]. The increased prevalence of OSAS appears to be related to obesity and male sex rather than to hypothyroidism per se [101]. However, decreased ventilatory responses [102], extravasation of albumin and mucopolysaccharides in the tissues of the upper airway [103, 104], and hypothyroid myopathy [99] have been suggested as possible contributing factors for OSAS in hypothyroidism.

In patients with OSAS, the prevalence of hypothyroidism is 1%–3% [100, 105], which is not different from that in the general population. 

In a group of obese patients with OSAS we have not found an impaired thyroid activity in basal conditions nor an altered TSH response to TRH challenge test [51], in agreement with some but not other studies. In fact, some derangement in TSH secretion in obesity with or without OSAS has been demonstrated [49, 56, 106]. 

As with other forms of systemic illness, suppression of thyroid responsiveness occurs during the development of OSAS with reversal of these changes during treatment [63]. Bratel et al. [56] have reported a more pronounced reduction of serum TSH in OSAS patients with the most severe nocturnal hypoxemia, with a normal TSH response to TRH before and after nCPAP treatment. However, TSH levels decreased even further after 7 months of nCPAP therapy [56]. In 101 overweight and obese male patients, Meston et al. [53] have described a small significant inverse correlation between OSAS and free T4 levels but not TSH, with no apparent association between obesity and either hormone. One-month nCPAP treatment compared to placebo resulted in a significant reduction in TSH without elevation in free T4 levels, consistent with the pattern of recovery from non-thyroidal illness. 

Biochemical screening for hypothyroidism in patients with OSAS is not considered necessary by many authors unless the patient is symptomatic or belongs to a risk group [105, 107, 108]. However, given the overlap in clinical presentation of primary OSAS and hypothyroidism, some authors indicate that screening for hypothyroidism is required to prevent misdiagnosis and that it is a cost-effective component of the investigation of sleep apnea [109].

Contrasting data are available concerning the efficacy of thyroid replacement therapy in improving sleep apnea in patients with clinical hypothyroidism. Some authors describe a prompt reversal of symptoms, sleep-disordered breathing, and nocturnal hypoxia [109] while little or no improvement in sleep apnea is reported by others [108].

### 3.5. Gonadal Axis

Among endocrine disturbances, changes of gonadal axis are common in patients with OSAS, who frequently show a hypogonadotropic hypogonadism likely due to alterations of the hypothalamic-pituitary control of gonadotropin synthesis and release. In particular, decreased morning and nocturnal testosterone concentrations have been found in lean and obese male patients with OSAS [49, 64–66] with an increase after uvulopalatal resection [65] or normalization after nCPAP treatment [49, 67]. Changes in sleep efficiency and architecture have been associated with alteration in pituitary-gonadal function in healthy older men [110, 111]. In young adults, the sleep-related rise in testosterone has been linked with the first rapid eye movement (REM) sleep episode and has been shown to be dependent on the integrity of the sleep process [112]. 

Gonadotropin levels have been found reduced both basally and after gonadotropin-releasing hormone (GnRH) stimulation but only partially reverted by hypoxia correction [49, 53]. Reduced Sex Hormone-Binding Globulin (SHBG) concentrations coupled to low testosterone levels and correlating to OSAS severity support a diagnosis of secondary hypogonadism [49, 53]. SHBG levels have been reported to rise during active nCPAP treatment [49, 53].

A significant correlation between LH/testosterone profiles and the severity of OSAS is described, thus suggesting that sleep fragmentation and hypoxia in addition to the degree of obesity may be responsible for the central suppression of testosterone in these patients. Moreover, testosterone concentrations fall with prolonged physical stress, sleep deprivation, and sleep fragmentation in normal young and elderly males [111–113]. Finally, hypoxia decreases LH and testosterone levels and alters circadian rhythm of testosterone secretion [24, 68, 114]. It has also been hypothesized that decreased testosterone levels may be part of an adaptive homeostatic mechanism to reduce sleep disordered breathing assuming that testosterone aggravates it [49, 115]. In fact, androgen levels can directly influence the prevalence and severity of sleep-disordered breathing and some reports have demonstrated that administration of exogenous androgens to both men and women can induce or precipitate apnea [116, 117]. However, few studies have systematically evaluated the effects of exogenous androgen replacement therapy on OSAS. Testosterone replacement therapy induced OSAS in one of five males and aggravated a preexisting sleep disordered breathing in another [117]. In 11 hypogonadal males, testosterone replacement increased apneic events but only in three subjects was the increase considered statistically significant [118]. In a placebo-controlled study of 17 overweight elderly males with partial androgen deficiency, testosterone replacement therapy decreased total sleep time and sleep efficiency and aggravated sleep apnea [119]. 

The few available data from females with sleep disordered breathing support the link with androgens. Irrespective of the menopausal state, obese females have higher androgen levels than nonobese females [69, 70]. In a lean, 70-year-old woman, a testosterone producing tumour caused sleep apnea, which disappeared after removal of the tumour [120]. 

Female hormones are thought to protect women from OSAS until menopause [121]. In clinical studies, male  :  female ratio of OSAS is ~10  :  1 [4, 122]. Among females referred to a sleep clinic, 47% of the postmenopausal and 21% of the premenopausal females had sleep apnea [123]. In a large community-based study, 1.9% of postmenopausal females and 0.6% of premenopausal females had OSAS, defined as AHI ≥10 and occurrence of daytime symptoms [6]. 

Menopause-related changes in body fat distribution, from gynoid phenotype to android features, may include deposition of fat around the upper airway [124]. The effect of menopause on body fat distribution is ascribed to declining levels of estrogen and progesterone and appears to be reversed or attenuated by the use of replacement hormones [125]. Declining levels of these hormones might also predispose some women to sleep-disordered breathing by lowering the ventilatory drive to the upper airway, leading to an imbalance between the collapsing forces of the upper airway during inspiration and the counteracting forces of the upper airway dilating muscles.

## 4. Conclusions

OSAS is a serious, prevalent condition which is strongly associated with obesity and has significant mortality and morbidity when untreated. Sleep fragmentation and hypoxia are likely to play a prevalent role in causing cardiovascular alterations that increase morbidity and mortality in comparison with simple obesity. The same factors can also be responsible for the endocrine abnormalities in OSAS that are frequently more marked than those in nonapneic obese patients. These abnormalities may be considered as adaptive changes which indicate how a local upper airway dysfunction induces systemic consequences. On the other hand, the same abnormalities can also contribute to the maintenance or progression of OSAS itself.

The recognition and understanding of the complex interactions between hormones and OSAS may allow a multidisciplinary approach to obese patients with this disturbance. Effective assessment and management of OSAS in obesity may correct endocrine changes, improve quality of life, and prevent associated morbidity or death.

## Figures and Tables

**Table 1:** Main hormonal changes in obesity and obstructive sleep apnea syndrome (OSAS).

	OBESITY without OSAS	OBESITY with OSAS	Reference
GH	↓	↓ ↓	[46–48]
IGF-1	n or ↓	↓ ↓	[48–50]
PRL	↓	n or ↑	[49, 51–53]
ACTH	↑	↑	[51, 54, 55]
Cortisol	↑	n or ↑	[49, 53–60]
Aldosterone	↑	↑	[61, 62]
fT_3_	n	n	[51]
fT_4_	n	n	[49, 51]
TSH	n	n or ↓	[49, 51, 56, 63]
LH	n or ↓	n or ↓	[49, 56, 64]
FSH	n or ↓	n or ↓	[49]
Testosterone in ♂	↓	↓	[49, 65–68]
Free Testosterone in ♂	n or ↓	↓	[49]
Testosterone in ♀	↑	?	[69, 70]
SHBG	↓	↓	[49]
Insulin	↑	↑↑	[11, 31–33]
Leptin	↑	↑↑	[35, 37–40]
Adiponectin	↓	↓ ↓	[36, 37, 42, 43, 71]
Ghrelin	↓	?	[39, 45]

n: normal; ↑: increased levels; ↓: reduced levels.
